# A Case of Cardiac Arrhythmia

**Published:** 1908-05-23

**Authors:** Leonard Williams

**Affiliations:** Physician to the French Hospital, Assistant Physician to the Metropolitan Hospital.


### Hospital Clinics.

### A CASE OF CARDIAC ARRHYTHMIA.

By LEONARD WILLIAMS, M.D., M.R.C.P., Physician to the French Hospital, Assistant Physician to the Metropolitan Hospital.

(A Clinical Lecture delivered at the Medical Graduates' College.)

This man, whose age is thirty-one, came to the Metropolitan Hospital early in January last complaining of palpitation of the heart. He is a sawyer by trade, and his work in the saw-mills has been both heavy and prolonged. Fifteen years ago he had an attack of rheumatic fever, and five years ago he had a second attack. When he first came to the hospital he was breathless, rather cyanotic, and his ankles were slightly swollen. These however were not the symptoms for which he sought relief. His complaint then was, and still is, that he is much troubled by palpitation of the heart. By palpitation of the heart is meant an inconvenient consciousness of having a heart, an obtrusiveness on the part of the organ which will not allow its existence to be forgotten. This state of matters is one which we are often content, at first at any rate, to assign to some cause outside the heart. Dyspepsia will frequently provoke it, especially when accompanied by flatulence; so will a sudden emotion, a sudden violent exertion, a rapid change of posture. The symptom is in fact due as a rule not directly to the heart itself, but to the nervous system acting on the heart. It is a cardiac manifestation of a nervous disturbance. Such, however, is by no means always the case, and we must be careful to remember that palpitation may be the only complaint of a person who is the subject of serious organic cardio-vascular disease. More especially is this true of aortic regurgitation. The large heart, the " cor bovinum " which accompanies that condition, is very apt unduly to emphasise its existence and punctuate its periods; and the enlarged left heart of renal cirrhosis not infrequently does the same. We should be careful then not to allow ourselves to be carried too far by the saying that a person who complains of his heart has probably nothing the matter with it. In so far as that saying is correct, it refers to palpitation which is transient and simple; it does not, and must not be allowed to, include palpitation accompanied by other cardiac symptoms, more especially if the palpitation, instead of being transient or paroxysmal, is persistent.

When this patient came to us in January, his whole appearance was so suggestive of a morbus cordis that I felt sure there was something more the matter with him than a mere nervous disturbance. On palpating his præcordial area, I was immediately encountered by the most significant and perhaps the most instructive of all the facts which proclaim a failure of compensation such as this man was evidently suffering from. This was the presence of a very pronounced degree of arrhythmia. Now the presence of arrhythmia tells us unmistakably of two things. One is that the excitability of the myocardium is increased (in the modern barbarous phraseology it is called a " positive bathmotropic influence "); the other is that the mitral valve is incompetent. Given, then, the hyper-excitability, the " irritable weakness " of the myocardium which is necessarily present in all cases of failing compensation, you may take it as a rule which has few, if any, exceptions, that the presence of arrhythmia proclaims that the mitral valve is incompetent. It does not mean that mitral regurgitation constitutes the only disorder present; on the contrary this condition more frequently than not is complicated by mitral stenosis: but it does mean, that whatever else may be wrong with the heart, this at any rate is happening, that each systole causes blood to regurgitate from the left ventricle into its corresponding auricle.

The train of events is as follows. We know that, normally, the wave of contraction is generated or provoked at the venous ostia, and that it passes from auricle to ventricle along the intra-ventricular bundle of His. When the condition of the auricular musculature is that of slight dilation coupled with hyper-excitability, which is called " irritable weakness," the contractions are more easily provoked. Accordingly, a reflux through the mitral opening, which in normally compensated states produces no stimulus, is sufficient, in failing compensation, to produce a very decided stimulus, with the result that each ventricular systole, by sending blood back into the auricle, provokes the latter into generating a contraction out of its turn, so to speak. The contraction wave is duly passed along, but the cavities are not empty; there has been no proper refractory period, and irregularity or arrhythmia results.

The presence of his arrhythmia, then, was sufficient to convince me that this man had mitral regurgitation, but when I came to examine him more carefully I became equally convinced that he was also the subject of mitral stenosis. To this conclusion I was forced by the fact that, in addition to the systolic bruit which was audible all over the mitral area, there was a diastolic murmur to be heard at one spot only, close to the apex. From the point of view of prognosis, this was an important and rather sinister finding. Failing compensation in uncomplicated mitral regurgitation, in a man of thirty, who has recently been subjected to hard physical labour, is not necessarily, or even usually, a very serious matter; but when the patient is shown to be the subject of mitral stenosis as well, the outlook becomes distinctly disquieting. Many people with mitral stenosis reach the age of thirty without ever having had the slightest cardiac trouble. But mitral stenosis is unfortunately a progressive disease, and it is about the age of thirty that it begins to produce unpleasant symptoms.

In order that his failing compensation, his " hypo-systole " as the French call it, might be properly treated, the patient was immediately sent into the wards, where he remained for six weeks.

During his stay he became very much better in every respect save that for which he originally applied for relief, namely palpitation. His palpitation and his arrhythmia have both persisted in spite of the varied and resourceful treatment to which my colleague Dr. Thursfield subjected him. In addition to digitalis, he was tried with strophanthus, convallaria, caffein, strychnine, and, to eliminate the nervous element, with bromide of potassium, but, so far as his arrhythmic palpitation is concerned, all to no purpose. Now, why is this? Why is it that the heart being no longer in a state of irritable weakness, the rhythm remains as irregular as ever? The reason is, I fear, to be found in the fibrous degeneration of the wall of the left auricle, which has been described as commonly present in all cases of persistent arrhythmia by several observers, notably by Radasewski* and by Merklen.† The patient, remember, has had a progressive narrowing of his mitral orifice for fifteen years; by working hard in the saw-mills, he has tried his circulatory system "high," as the saying is; and it is certain that the brunt of this extra work has fallen upon the left auricle, which has thus been subjected to far more than its fair share of pressure.

Now, we know that one of the results of undue pressure in the arteries is fibrous degeneration of the muscular walls. The heart is structurally little more than a glorified artery, and it is reasonable to believe that the same forces acting on the former will occasionally produce the same effects as we know them to produce in the latter. For fifteen years then, this man has suffered from "high tension" in his left auricle, due partly to his mitral regurgitation and partly to his mitral stenosis. During the latter portion of that period a process of fibrous degeneration has been going on in the walls of the chamber, and we now find him with a persistent arrhythmic palpitation against which drugs have been appealed to in vain. The arrhythmia is due, as I have already explained, mainly to the extra systoles provoked in an irritable myocardium by the regurgitant stream. The fact that it is persistent, that it declines to yield with the other signs of failing compensation, is due to fibrous degeneration of a portion of the auricular musculature, thereby reducing the remaining portion to a chronic state of "irritable weakness." Reduced to its simplest terms then, the case is one of rheumatic endocarditis commencing at fifteen years of age, and aggravated by a second attack five years later; subjected to a considerable degree of cardio-vascular strain by work in a saw-mill for several years; ending in a breakdown at thirty-one years of age, of which the prognosis is at the best, gloomy.

So far as the treatment of such a case is concerned, the immediate necessities will be best met by iodides, caffein, and bromides, though it must be confessed that such good as they may effect will not be very prolonged. Could we have caught this patient at, or soon after, his first rheumatic attack, we might very easily so have ordered matters as to obviate his present "impasse." Had he been well off, we should have sent him to Bourbon-Lancy, a station which, in the treatment of the effects of endocarditis in rheumatic children, is too much neglected by English physicians, a neglect which is the less comprehensible on account of the unfavourable outlook with which such cases are necessarily otherwise regarded. But whether or not spa treatment was feasible, by prohibiting this patient from engaging in heavy manual labour, we should have prevented the constant strain upon the cardio-vascular system which has been the direct, the proximate, cause of his persistent arrhythmic palpitation, the clinical expression of a fibrous degeneration of the walls of his left auricle, due to continued high tension within the chamber, a state of matters for which there is obviously no remedy.

* "Ueber die Muskelerkrankungen der Vorhöfe der Herzens" ("Zeitshrift f. Klin." Med. XXVII., p. 381).

† "Comptes Rendus," du 5, Congress de Med. France, Lille, 1899.

